# Seroprevalence of Transplacentally acquired Measles antibodies in unvaccinated infants at nine months of age and its relation to the feeding practices

**DOI:** 10.1186/s12879-019-4167-0

**Published:** 2019-07-05

**Authors:** Najma Javed, Muhammad Arif Nadeem Saqib, Mir. Muhammad Hassan Bullo, Rana Jawad Asghar

**Affiliations:** 1 Central Research Centre, PHRC, National Institute of Health, Islamabad, Pakistan; 2 Pakistan Medical Research Council, Islamabad, Pakistan; 3 Federal General Hospital, Islamabad, Pakistan; 4 Field Epidemiology and Lab. Training Program, Islamabad, Pakistan

**Keywords:** Measles, Infants, Vaccination, Antibodies, Feeding practices

## Abstract

**Background:**

In recent years Pakistan has faced frequent measles outbreaks killing hundreds of children despite the availability of vaccine for decades. This study was undertaken to determine the persistence of maternal transferred measles antibody levels in infants before measles vaccination with relation to their feeding practices.

**Methods:**

A cross sectional study was conducted at district Islamabad over 1 year between 1st October 2013 to 30th Sept. 2016. Any infant less than 9 months of age, not suffering from an acute or debilitating illness and not vaccinated was enrolled in the study. After taking written informed consent from parents / guardians, information was collected on a pretested questionnaire. About 3 cc venous blood was taken to quantify any measles IgG antibodies. Data was analyzed by using Epi Info 7.2 version.

**Results:**

Three hundred eighty-four infants were enrolled and were divided into three age groups, 1–90, 91–180 and 181–270 days age groups. Mean age of infants was 4.4 months ±3.2 SD. Male to female ratio was 1.2:1. A level of maternal measles IgG antibodies ≥12 U/ml was taken as protective. Of total 384 infants, 91(24%) had protective measles antibody titters (> 12 U/ml). and 65 (73%) of them were on breast milk. Highest antibody levels were found in 1–90 days age group. Analysis showed that 181–270 days aged infants had 3.1875 more odds of having unprotected/ low levels of antibodies against measles than children aged less than 180 days. Age group < 180 days found to be statistically significant with protective IgG levels (OR: 3.1875, *P* value: < 0.000063).

**Conclusion:**

Measles protective antibodies were found in infants < 180 days age group. Breast feeding provides early protection against measles. Levels drop down to low levels immediately after birth and then after 06 months. It is, therefore, recommended that measles vaccination should be considered for administration at 6 months or even earlier if measles immunity is desired.

## Background

Measles is one of the most common childhood fevers which is highly contagious and characterized by coryza, cough, conjunctivitis and specific exanthema followed by generalized maculo-papular eruption [1]. Transmission of virus is probably through secretions shed from the respiratory tract during the prodromal phase and early stages of rash when cough and coryza are intense [2]. According to World Health Organization (WHO), during the year 2015, globally 367 deaths/ day occurred due to measles in children under 5 years of age [3]. Morbidity and mortality related to measles frequently occurs before 9 months of age [4]. However, vaccination against measles is recommended at the age of 9 months in developing countries.

In Pakistan measles vaccination coverage has increased and its reported first dose coverage reached 88% in 2012 [5]. However coverage for the second dose was only 53%. According to WHO, this coverage is not sufficient to prevent outbreaks [6, 7]. In December 2012, measles outbreaks started in Sind province and lasted until August, 2013 and killed 321. In the first half of 2013, 94 cases were diagnosed in Punjab province and majority of deaths occurred in Lahore, Capital city of Punjab [8]. Primary protection against infectious diseases at birth is provided mainly by maternal antibodies [9]. A number of factors affect maternal antibody concentration in young infants. Duration of gestation determines the placental transfer of antibodies to the newborn: that is the reason, preterm babies receive significantly fewer antibodies [10].

The sources of maternally transmitted antibodies [11] in children are those transferred in utero via the placenta and the colostrum (breast milk). The estimated duration of protection by maternal antibodies for different diseases varies among infants. Protective antibody levels are though to last until: 3.3 months for measles, 2.7 months for mumps, 3.9 months for rubella, and 3.4 months for varicella.

This passive immunity to measles, mumps and rubella usually lasts for about a year [12]. About 13% of the current under five mortality rates could be averted by promoting the proper breastfeeding practices [13].

Considering these facts this study was undertaken to determine specific antibodies (IgG) against measles to evaluate the immune status of the under 9 months aged children before the start of immunization to provide evidence to policy makers regarding the measles vaccination schedule.

## Methods

### Study design

A cross sectional community based survey was conducted in Islamabad, Pakistan over 12 months (1st Oct. 2015 to 30th Sept. 2016). Cluster sampling technique was used for selection of study site. In the first phase Islamabad was divided into urban and rural strata and then six clusters each from urban and rural strata were selected.

For urban; list of sectors was obtained and only three sectors were selected while in case of rural cluster, three villages/ suburban areas were selected by convenience sampling however the households were selected randomly. Rural population is defined as people living in rural areas as determined by national statistical offices of Pakistan and it is calculated as the difference between total population and urban population excluding army. Fifty samples were collected from each locality.

### Inclusion criteria

Healthy infants aged < 09 months of either gender with informed consent of participation by their parents / guardians.

### Exclusion criteria

Children with the recent history of an acute illness/ chronic illness such as tuberculosis, hepatitis etc. and children suffering from laboratory confirmed measles illness.

Sample size: Sample size was calculated on the basis of population under 1 year in 2012 (24.3 births/ 1000 population in midyear or 4 million/ year) with 5% margin of error, 95% confidence interval and with 10% refusal rate as 384 infants.

## Methods

### Ethical considerations

The study had been ethically approved by Institutional Review Board (IRB) of Pakistan Medical Research Council (PMRC), Islamabad.

The Lady Health Workers (LHWs) of selected areas were used as community gate keepers. A lady health worker is a female who has secondary level of education. She is selected from the same community where she will serve after training by the health department. She is a paid worker and works within the community, collects data about different diseases and shares this data on a monthly basis to her supervisor. LHWS in the selected study area helped the team in their communications about the study starting a day before survey.

The research team consisting of one male, one female data collector and a phlebotomist. They started identifying houses from periphery and worked systematically towards the center of the selected area - which was a mosque or common sitting place for villagers in rural areas and in case of urban sectors, it was central market “MARKAZ”. After arriving at the periphery, the right or left lane was selected by tossing a coin and then the first house was selected randomly. Every second house was knocked to enquire about the presence of child as per inclusion criteria. If the door was locked or child was not found or in case of refusal, the next second house was selected. Within the selected house, in case of more than one eligible child, children were selected randomly.

The questionnaire was formulated and shared with subject experts for construct and conduct validity. Suggestions were incorporated and field testing was done. Feedback received after pretesting was also incorporated in final data collection tool. The final questionnaire had three sections: demographic information of the mother and the child, history of duration of pregnancy and mode of delivery, feeding practices of the newborn and third sections about measles vaccination. Written informed consent was taken from parents/ guardians, before starting recording the information on the questionnaire.

Blood samples (3.0 cc) were drawn in gel tube from all study participants and was labeled. Samples were centrifuged and sera were analyzed to detect Measles antibodies (IgG) using ELISA. The ELISA Measles IgG titer of ≥12 U/ml was considered “protective” or “positive.” whereas titers below 12 U/ ml were reported as “unprotective” or “Negative”. In this study, we expressed the measles IgG in U/ml according to the reference provided by the ELISA kit manufacturer, rather than in milli-international units per milliliter based on the international standards. Simple criteria based on the detection limit of < 0.00 for measles antibody was used to exclude low concentration data (relative variance).

### Test procedure

The serum samples were tested for the measles IgG antibodies. Technique: Measles IgG antibodies were tested using ELISA kits at central Research Centre, PMRC, NIH, Islamabad.

### Statistical analysis

We analyzed data using Epi Info version 7.2. The percentages of positive and negative sera were calculated. Means were computed for age and IgG titers. For testing the association between continuous variables, multiple linear regression was used.

## Results

Three hundred eighty-four infants were enrolled. Once the infants were enrolled, participants were divided into three age groups and included 1–90 days, 91–180 days and 181–270 days age groups.

Mean age of infants was 4.4 months ±3.2 SD. Male to female ratio was 1.2:1 whereas mean maternal age was 26.8 ± 4.9 SD. A level of maternal measles IgG antibodies ≥12 U/ml was taken as protective as well as positive for presence of measles antibodies after vaccination. Mean titer of IgG antibody was 0.57 U/ml. Of total 384 infants, 91(24%) were found to have protective measles antibody titers (> 12 U/ml), remaining 293 (76%) infants had lower levels. Out of 91infants who had protective measles antibodies, 65 (73%) infants were on breast milk, (Table 1). Among mothers, only 09 were vaccinated. Results showed, out of 09 infants of immunized mothers, only two infants were found to be positive for measles antibodies while rest had no antibodies.

**Table 1:** Presence of protective maternal Measles IgG antibodies based on feeding practices among study population

Feeding groups	Total	Protective Antibodies present	Protective Antibodies absent	Mean IgG In U/ml
Breast feeding	267	65 (24%)	203 (76%)	0.574
Animal milk	24	4 (17%)	20 (83%)	0.466
^a^Formula/supplement	64	18(28%)	46 (72%)	0.634
^b^Others	29	4 (14%)	25 (86%)	.517
TOTAL	384	91	293	

^a^Powdered Formula

^b^(using mother milk, animal milk, formula together) Infant is taking milk from more than one category

Out of 483, 71.8% (275) were of term gestation and delivered normally while 106 were delivered through C-Section with mean IgG values of .569 and .596 respectively.

### Analytical epidemiology

Based on current study findings, the odds of contracting measles is 1.26 times greater in children aged 6–9 months than children less than 6 months of age. However, it should be noted that transmission, or contracting measles, is complex and potentially requires lack of herd immunity, exposure to measles, nutritional status, vaccine technique, unprotective titers at birth, etc. Table 2.

**Table 2:** 2 × 2 table

Age groups	Unprotected IgG Levels	Protective IgG levels
6–9 months	119	16
1–6 months	174	75
	Odds ratio: 3.187 (CI 1.7–5.7) *P* value: < 0.000063	

Out of 384 participants, there were 143 (37%), 108 (28%) and 134 (35%) infants in age groups of 1–90, 91–180 and 181–270 days respectively. Mean IgG levels in the 1–90 days group was > 8.0 U/ml, > 12 U/ml in 91–180 days group and > 8.0 U/ml in group 181–270 days age group whereas p. values were .0001, .608 and .484 respectively.

## Discussion

In this study we found that in the 1–90 days age group the mean measles IgG level was > 8.0 U/ml, in the 91–180 days group < 12 U/ml and in the 181–270 days age group it was > 8.0 U/ml with respective p. values of 0.0001 (significant), 0.608 (Not significant) and 0.484 (Not significant). Regarding the feeding practices, IgG antibodies were 0.574 (8-12 U), 0.466 (< 8.0 U) and 0.634 (> 12 U) in breast feed group, animal feed group and breast milk+ top feed group respectively. Out of 483, 71.8% (275) were of term gestation and delivered normally while 106 were delivered through C-Section with mean IgG values of 0.569 and 0.596 respectively which are not statistically significant. These findings are in accordance with other studies [14–16] revealing that the majority of maternal measles antibodies transfer across the placenta takes place during the third trimester of gestation and this transfer is receptor mediated. Another study added that preterm delivery was associated with lower but protective levels of Maternal Measles Antibodies (MMA) and post-term deliveries with higher levels of MMA than term deliveries. In our study 72% deliveries were at term however all normally delivered babies did not have protective levels of maternal measles IgG antibodies. It may be due to the fact that mother’s own immunity against measles (presence of IgG antibodies) also plays a role in transplacental transfer of antibodies to neonates.

This study revealed that the measles protective measles antibodies were present in 91–180 days age group as compared to 1–90 days age group and 181–270 age groups. Muscat et al. in their study discussed that there is waning of the maternal transferred measles antibodies in the infants before vaccination age e.g., < 1 year, therefore measles risk and severity are greater than the risk and severity among those aged ≥1 year [17]. It is consistent with our findings.

A Nigerian study revealed that 58% of children had lost the protective maternal antibody by the age of 4 months and only 3% of the children had enough antibodies to protect them between the ages of 6–9 months [18]. Our study has the same findings emphasizing the need for early measles vaccination to protect infants from becoming victim of this fatal illness.

Sandra et al. in their study found that the estimated duration of protection by maternal antibodies among infants was short e.g. 3.3 months for measles [19], and our results are the same. Another study [20] showed that the rate of decay of passive immunity was slower in infants of naturally immune women (un-vaccinated), but the median time to loss of immunity was longer than that in infants of vaccinated mothers (3.78 vs. 0.97 months). This is in contrast to our study in which out of 09 vaccinated mothers, only two had enough levels to transfer antibodies to their newborns. However, this is one of the limitations of our study that we did not follow the infants to record the decay of maternal antibodies in infants.

In Pakistan, not every child is born with enough trans-placentally transferred anti-measles antibody levels to protect the child during infancy. Further, most of the maternal acquired antibodies wane before reaching the current recommended age for vaccination e.g. 09 months. It is, therefore suggested that vaccine may need to be repeated (> 2 doses) and that the measles first dose is delivered before 09 months of age, if measles immunity is desired. It calls an urgent need to review the measles vaccination schedule from 09 months to 06 months to protect these susceptible infants.

## Conclusion

In our current study, measles protective antibodies were found mostly among infants < 180 days age group. Of total 384 infants, 91(24%) had protective measles antibody titters (> 12 U/ml) and 65 (73%) of them were on breast milk. This implies that breast feeding provides early protection against measles however, these acquired levels drop down to low levels immediately after birth and then after 06 months. In Pakistan, though women tend to breast-feed their babies, however due to poor maternal nutritional status coupled with repeated pregnancies; don’t allow mothers to nurse their babies with exclusive feeding. Secondly, measles is endemic in Pakistan so the current schedule to give first measles shot at the age of 09 months leaves the child vulnerable to this preventable illness. Based on the results of this study, we urge further consideration to the possibility of earlier measles vaccination administration, such as at 6 months, to attempt to save more infants from this fatal infection.

## Abbreviations

ELISA: Enzyme Linked Immunosorbent Assay
LHWs: Lady Health Workers
MMA: Maternal Measles Antibodies
WHO: World Health Organization
